# Retraction: Outcomes of Direct-Acting Antivirals Versus Interferon-Based Therapy in Chronic Hepatitis C Infection

**DOI:** 10.7759/cureus.r173

**Published:** 2025-04-01

**Authors:** Ratna Chowdhury, Wardah Rashid, Taranpreet Singh, Abdur Rehman, Nida F Daterdiwala, Varaidzo Mkosi, Bhumikala Limbu, Syeda Alliza Bukhari, Afif Ramadhan, Muath M Dabas, Abdullah Shehryar, Ramadan Khan

**Affiliations:** 1 Internal Medicine, Saint James School of Medicine, Arnos Vale, VCT; 2 Internal Medicine, Khawaja Muhammad Safdar Medical College, Sialkot, PAK; 3 Internal Medicine, Mahatma Gandhi Mission (MGM) Medical College and Hospital, Mumbai, IND; 4 Surgery, Mayo Hospital, Lahore, PAK; 5 Internal Medicine, Ziauddin University, Karachi, PAK; 6 Internal Medicine, Ternopil National Medical University, Ternopil, UKR; 7 Emergency Medicine, Bishweshwar Prasad Koirala Institute of Health Sciences, Dharan, NPL; 8 Internal Medicine, Jinnah Sindh Medical University, Karachi, PAK; 9 Internal Medicine, Universitas Gadjah Mada, Yogyakarta, IDN; 10 General Surgery, University of Jordan, Amman, JOR; 11 Internal Medicine, Allama Iqbal Medical College, Lahore, PAK; 12 Internal Medicine, Dera Ghazi Khan Medical College, Dera Ghazi Khan, PAK

The Editors-in-Chief have retracted this article. An investigation by the journal found evidence of authorship manipulation. The Editors-in-Chief therefore no longer have confidence in the provenance and reliability of the article contents. The authors agree with the decision to retract.

